# Changes in Dynamic Thiol/Disulfide Homeostasis, and Substance P, B-Endorphin and α-Tocopherol Concentrations in the Spinal Cord of Chronically Lame Dairy Cows

**DOI:** 10.3390/ani13101620

**Published:** 2023-05-12

**Authors:** Heine Müller, Daniel Herzberg, Ricardo Chihuailaf, Pablo Strobel, Marianne Werner, Hedie Bustamante

**Affiliations:** 1 Graduate School, Faculty of Veterinary Sciences, Universidad Austral de Chile, Valdivia 5110566, Chile; 2 Veterinary Clinical Sciences Institute, Faculty of Veterinary Sciences, Universidad Austral de Chile, Valdivia 5110566, Chile; ricardo.chihuailaf@uach.cl; 3 Veterinary Clinical Hospital, School of Agricultural and Veterinary Sciences, Universidad Viña del Mar, Viña del Mar 2571959, Chile; 4 Animal Science Institute, Faculty of Veterinary Sciences, Universidad Austral de Chile, Valdivia 5110566, Chile

**Keywords:** dairy cows, thiols, lameness, substance P, β-endorphin, α-tocopherol, pain

## Abstract

**Simple Summary:**

Chronic inflammatory lameness is a common painful condition in dairy cows that affects animal welfare and produces significant yield losses. Bovine chronic lameness can lead to a chronic pain condition due to the participation of pro- and anti-nociceptive mediators and reactive oxygen species. The results of this study revealed increased concentrations of substance P and β-endorphin in lame dairy cows, indicating chronic pain development and sustained endogenous analgesics release. Moreover, the results revealed decreased disulfide levels and α-tocopherol concentrations in the lame group, suggesting an overproduction of reactive oxygen species, which may indicate their involvement in the development of chronic pain in bovine lameness.

**Abstract:**

Initial lameness inflammation leads to chronic lameness and development of chronic pain due to the release of pro-inflammatory mediators such as reactive oxygen species (ROS), which are implicated in the transition from acute to chronic pain, and free radical scavengers countering thiol, substance P (SP), and β-endorphin (BE). The present study aimed to evaluate the dynamic thiol–disulfide homeostasis, α-tocopherol concentrations and SP and BE concentrations in the spinal cord of chronically lame dairy cows. Ten lame and 10 non-lame cows with a parity range of 2–6 were selected for the study. Lame cows had a history of up to 3 months of lameness. Spinal cord samples were obtained from the L2 to L4 lumbar vertebrae aspect of each animal. A thiol–disulfide homeostasis assay was performed using absorbance, and the α-tocopherol concentration was determined by HPLC. SP and BE concentrations were measured using ELISA kits. The results indicated that SP and BE were significantly higher in the spinal cord of lame cows. In contrast, disulfide levels and α-tocopherol concentrations were significantly lower in the spinal cord of lame cows. In conclusion, disulfide levels and α-tocopherol concentrations indicated a defective antioxidant response in cows with chronic lameness. The results of SP and BE concentrations suggested chronic pain and a defective endogenous analgesic response.

## 1. Introduction

Lameness, an abnormal gait that usually originates after injury, disease, or dysfunction of one or more feet and/or limbs, is an important and persistent problem that affects dairy cows worldwide, negatively affecting their welfare [1]. Lameness-associated pain results from tissue damage at the site of injury in which inflammation arises, initiating the release of multiple pain mediators and chemical substances that contribute to the painful process. Inflammation causes an increase in the concentration of chemical mediators in the damaged tissue that leads to further activation of peripheral receptors, leading to central and peripheral sensitization and the development of clinical entities, including hyperalgesia and allodynia [2,3]. Chronic lameness leads to chronic painful states and the presentation of decreased nociceptive thresholds, indicating hyperalgesia and allodynia [4].

Considerable evidence implicates reactive oxygen species (ROS) and reactive nitrogen species (RNS) in the development of chronic pain and the transition of acute to chronic pain [5], as they can modulate proteins kinases [6], alter glutamatergic neurotransmissions through increasing phosphorylation of the NR1 subunit of the N-methyl-D-aspartate receptor (NMDA) [7], induce neuroinflammation [8], and modulate ion channels such as the transient receptor potential cation channel, subfamily V, member 1 (TRPV1) [9]. All these changes contribute to the development of central sensitization associated with acute and chronic inflammatory and non-inflammatory neuropathic pain [5].

This strong tendency to produce oxidation by ROS is countered by antioxidant compounds such as thiols and free radical scavengers in order to maintain an adequate balance between ROS production and elimination [10,11]. Thiols react with ROS, producing molecular disulfide bonds [12], which can be reduced to thiols again in order to maintain the dynamic thiol–disulfide homeostasis [13]. Thiol–disulfide homeostasis is used as an image of the oxidative stress condition in various human diseases [14,15,16,17]. Furthermore, Vitamin E inhibits the production of ROS, acting as a free radical scavenger through the intervention of lipid oxidation and the glutathione peroxidase pathway [18,19,20]. A-tocopherol is chemically and biologically the most active isoform of vitamin E [21] that induces anti-inflammatory effects by inhibiting protein kinase C activity [22]. Moreover, it has been reported that vitamin E is able to reduce hyperalgesia and allodynia and to suppress neuropathic pain in models of sciatic crush nerve injury in rats [23]. 

Chronic pain and central sensitization have been associated with neuroinflammation triggered by neurotransmitter and pro-inflammatory mediator release such as glutamate and substance P (SP) in the dorsal horn of the spinal cord [24] after glial cell activation [25]. Release of SP in the dorsal horn activates glial cells, leading to the expression and release of inflammatory mediators [26], including nitroxidative species [24]. Moreover, it has been confirmed that SP represents an important upstream modulator of the endocannabinoid [27] and endogenous opioid signaling pathways [28], and that the NK1 receptor may play a critical role in pain-induced analgesia [29].

Additionally, the sensory information transmitted from peripheral nociceptors to the dorsal horn via afferent nerve fibers is modulated partially by β-endorphin (BE) [28]. Endogenous opioids are involved in the innate pain-relieving system via the activation of μ-, δ-, and κ- receptors [30], which are located on neurons, axons, and dendrites intrinsic to the spinal cord and on the terminals of primary afferents fibers [30,31]. Particularly, BE is released in order to modulate endogenous analgesic mechanisms through the interaction with the specific opioid μ-receptor located in the central and peripheral nervous system [32]. BE released via the peripheral and central systems is mediated by the pituitary and hypothalamic pro-opio-melanocortin neurons, respectively [33].

Based on previous studies from our group, we hypothesized that chronic inflammatory lameness in dairy cows is mediated by changes in the oxidative stress and the endogenous opioid responses in the dorsal horn of the spinal cord. Furthermore, we aimed to evaluate the dynamic thiol–disulfide homeostasis, α-tocopherol concentrations, and SP and BE concentrations in the spinal cord of chronically lame dairy cows.

## 2. Materials and Methods

### 2.1. Animals

A total of 20 animals, including 10 Holstein and 10 Kiwi cross dairy cows with a parity range between 2 and 6 and weighing between 350 and 450 Kg, were used in this study. Lame cows were selected according to the following criteria: history of up to 3 months of lameness in a rear limb caused by chronic white line disease, sole hemorrhage, and sole ulcer. Exclusion criteria for both groups included the presence of neurological diseases, acute or chronic mastitis, reproductive or respiratory disorders, and any other systemic disease including metabolic and mineral disorders. Control animals (*n* = 10) were selected from a local slaughterhouse after official consent by the sanitary authority according to the following criteria: No history on lameness in the last 3 months and a mobility score of 0 in a clinical examination [34]. Lame cows were euthanized after intravenous general anesthesia by administering intrathecal lidocaine in the atlanto-occipital foramen. Non-lame cows were slaughtered at an abattoir by mechanical stunning and exsanguination according to national regulations and guidelines following the World Organization for Animal Health guidelines. The researchers were not involved in the decision for euthanasia or slaughter.

### 2.2. Lameness Assessment

Animals were grouped into lame (*n* = 10) and non-lame cows (*n* = 10). A complete clinical examination was performed in all enrolled cows based on a detailed visual inspection, pain reaction to palpation and evaluation of the integrity of affected tissues. Lameness was confirmed and classified by the farm veterinarian according to the mobility score previously described by Reader and colleagues (2011) [34]. Additionally, all lame cows belonged to a flock of lame cows that was diagnosed and treated by a veterinarian podopathologist.

### 2.3. Spinal Cord Processing, Protein Extraction, and Quantification

After euthanasia, spinal cord samples were obtained after carefully removing the dorsal aspect of the L2 to L4 lumbar vertebrae following the procedure described by [3]. A segment of 20 cm of lumbar spinal cord was obtained from each animal. After gently dissecting, the dura mater and arachnoid were removed, and spinal cord tissue samples were snap-frozen in liquid nitrogen. Segments of 250 mg of dorsal horn spinal cord tissue ipsilateral to the lesion were allocated into a mix of 1 mL of phosphate buffered saline (PBS) with 10 μL of protease inhibitor. The samples were then homogenized in 1 mL of PBS using an Ultra Turrax tissue homogenizer (T10, IKA^®^, Staufen, Germany) at 4 °C and 16,000 rpm three times for 30 s each. All samples were immediately sonicated for 30 s and centrifuged at 20,000 g for 10 min, and the supernatant was removed, collected, and frozen. Protein quantification was performed using the Pierce^®^ bicinchoninic acid (BCA) protein assay kit (Thermo Scientific, Rochford, SD, USA).

### 2.4. Spinal Dynamic Thiol/Disulfide Homeostasis Assay

Determination of dynamic thiol/disulfide homeostasis was performed according to the method described by Erel and Nesselioglu (2014) [14]. Native thiol and total thiol content were synchronously measured as a paired test. In a single well, native thiol groups were measured. At the parallel run, dynamic disulfide bonds were reduced to free thiol groups, and then total thiol was measured. Briefly, the assay for total thiols was performed by adding 10 μL of R1 (NaBH4), 10 μL of sample, and 110 μL of R2 (formaldehyde/EDTA/Tris) sequentially to the vessel, and the first absorbance was measured. Subsequently, 10 μL of R3 (DTNB) was added to the vessel, and after 10 min, the second absorbance was taken. The assay for native thiols was performed similarly as previously described for total thiols, replacing R1(NaBH4) for 10 μL R1’(NaCl). Absorbances were read at 415 nm using a microplate reader (PHOmo, Autobio Labtec Instruments CO., Ltd., Zhengzhou, China). Final absorbance for total thiols was obtained by subtracting the first average from the second average absorbance for each sample. The same process was used for native thiols’ final absorbance. Concentrations of total and native thiols was obtained by correcting absorbances with protein concentrations of each sample. The disulfide parameter value was calculated as half of the difference of the total thiols and native thiols. Thiol/disulfide homeostasis was determined by calculating the ratio between disulfide and native thiol concentrations and was expressed as spinal disulfide levels. All the experiments were performed in triplicates for each sample and replicated three times. The totality of the results values was included for final analysis. The coefficients of variation inter- and intra-assay were 7.91% and 3.63%, respectively.

### 2.5. Spinal Substance P and β-Endorphin Immunoassay

β-endorphin concentrations were measured using an ELISA kit (#RD-bEP-b, Red Dot Biotech Inc., Kelowna, BC, Canada) with a detection range between 15.6 and 1000 pg/mL and a minimum detectable concentration of 4.89 pg/mL. Briefly, 50 uL of each sample was loaded in duplicate, incubated, and read at 450 nm using a microplate reader (PHOmo, Autobio Labtec Instruments CO., Ltd., Zhengzhou, China). Similarly, SP concentrations were measured using an ELISA kit (#KGE007, R & D systems Inc., Minneapolis, MN, USA) with a detection range between 78.1 and 2500 pg/mL and a minimum detectable concentration of 16.8 pg/mL. Briefly, 50 uL of each sample was loaded in duplicate, incubated, and read at 570 nm using a microplate reader.

### 2.6. Determination of α-Tocopherol Concentration by High-Performance Liquid Chromatography (HPLC)

For determination of α-tocopherol concentrations, a protocol proposed by Chihuailaf et al. [35] was used with modifications. Briefly, 1 mL of homogenate of each sample was taken in a test tube under indirect light. Then, 100 μL of tocopherol acetate 500 ppm (Sigma) was added as an internal standard. The lipids were extracted by addition of 1.5 mL of ethanol HPLC grade (Merck) and 2 mL of cyclohexane HPLC grade (Merck). Each sample was vortex-mixed for 60 s, and then all samples were centrifugated at 2000× *g* for 10 min. The cyclohexane top layer was taken and added in a 4 mL vial. The vials were placed in a water bath at 35 °C to evaporate thee supernatant under a nitrogen stream. Finally, the dry samples were reconstituted with 100 μL of an ether–ethanol solution (10%) and then shaken for resuspension. All samples were submitted to HPLC for determination of α-tocopherol concentrations in an HPLC Shimadzu equipped with a diode-array detector and a C18 column (150 × 4.6 mm × 4.5 μm). The chromatographic condition was isocratic, using methanol as a mobile phase delivered at a flowrate of 2 mL/min. The detection of α-tocopherol was at 294 nm and internal standard at 285 nm. The α-tocopherol concentration was calculated from a calibration curve adjusted previously to the recovery of the internal standard and expressed in micrograms per milliliter. The concentrations of the standard curve were 0.5–1–5.932–11.864–23.728–41.524 ppm, and the lower detection range was 0.5 ppm.

### 2.7. Statistical Analysis

For each outcome variable, normality was evaluated with the Shapiro–Wilk test. Differences of spinal cord disulfide levels and substance P, β-endorphin, and α-tocopherol concentrations between lame and non-lame groups were evaluated using an unpaired *t*-test. A *p*-value lower than 0.05 was considered significant. Statistical analyses were performed in GraphPad Prism 9.0, version 9.5.1 (GraphPad Software, San Diego, CA, USA).

## 3. Results

### 3.1. Substance P Spinal Cord Concentrations

**Figure 1:**
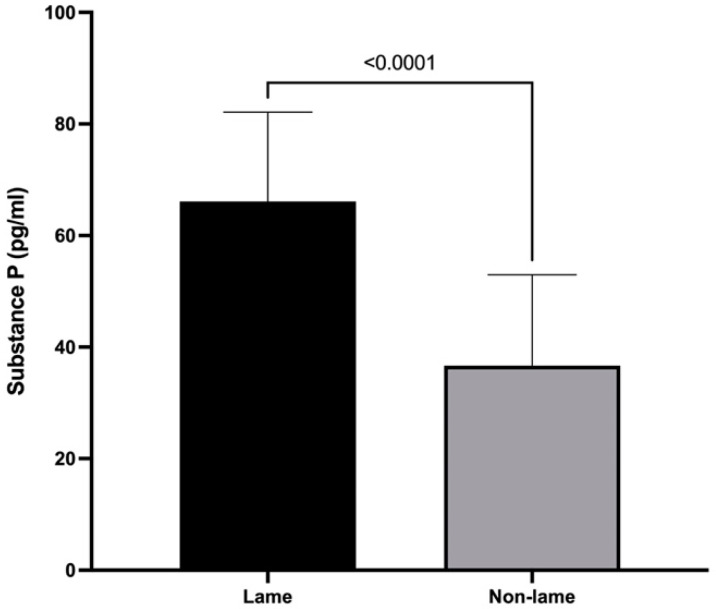
Mean + SD spinal cord substance P concentrations of chronically lame and control dairy cows.

Lame cows had a mean spinal cord concentration of SP of 66.1 ± 16.0 pg/mL, and non-lame cows had a concentration of 36.67 ± 16.13 pg/mL (*p* < 0.0001) (Figure 1).

### 3.2. β-Endorphin Spinal Cord Concentrations

**Figure 2:**
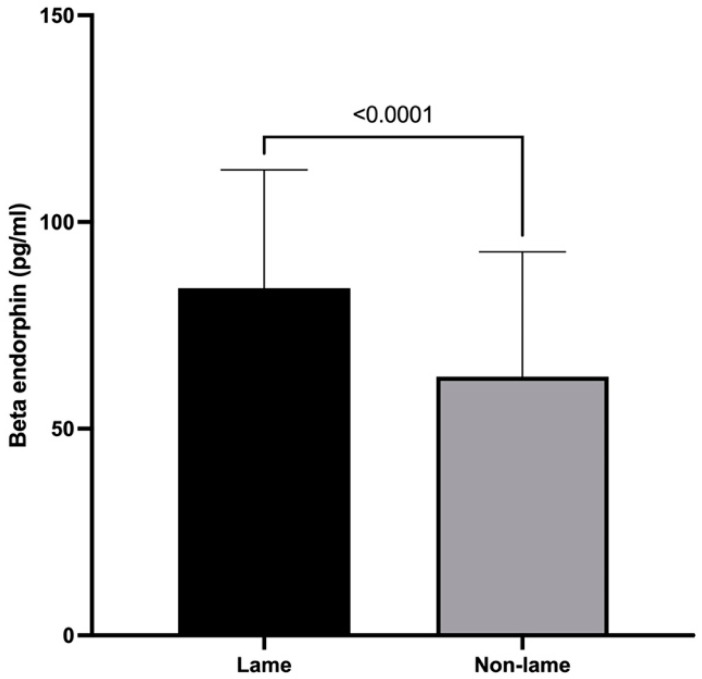
Mean + SD spinal cord concentrations of β-endorphin of chronically lame and control dairy cows.

The mean spinal cord concentration of BE was 84.0 ± 28.60 pg/mL in the lame group and 62.61 ± 30.16 pg/mL in the non-lame group (*p* < 0.0001) (Figure 2).

### 3.3. Dynamic Thiol/Disulfide Homeostasis

**Figure 3:**
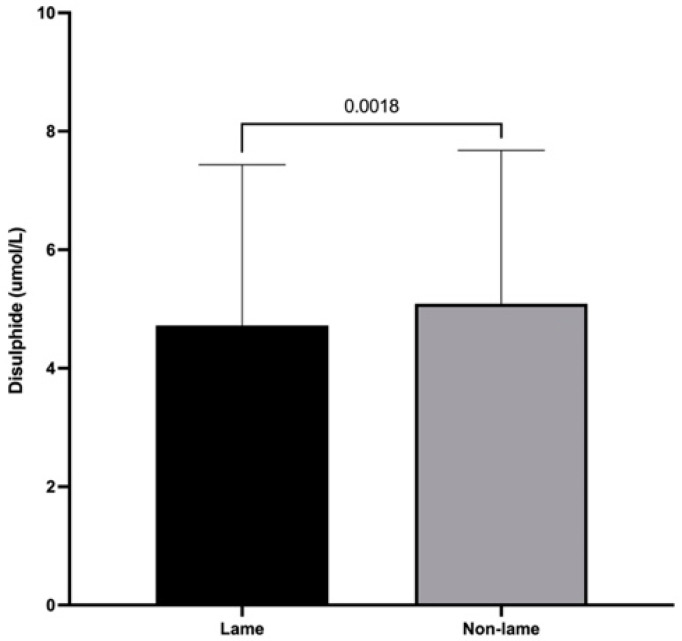
Mean + SD for spinal cord disulfide levels of chronically lame and control dairy cows.

Mean spinal cord disulfide levels were 4.72 ± 2.71 and 5.09 ± 2.59 μmol/L for lame and non-lame cows, respectively. Lame cows had significantly decreased disulfide spinal cord levels compared to those of non-lame cows (*p* < 0.01) (Figure 3).

### 3.4. α-Tocopherol Spinal Cord Concentrations

**Figure 4:**
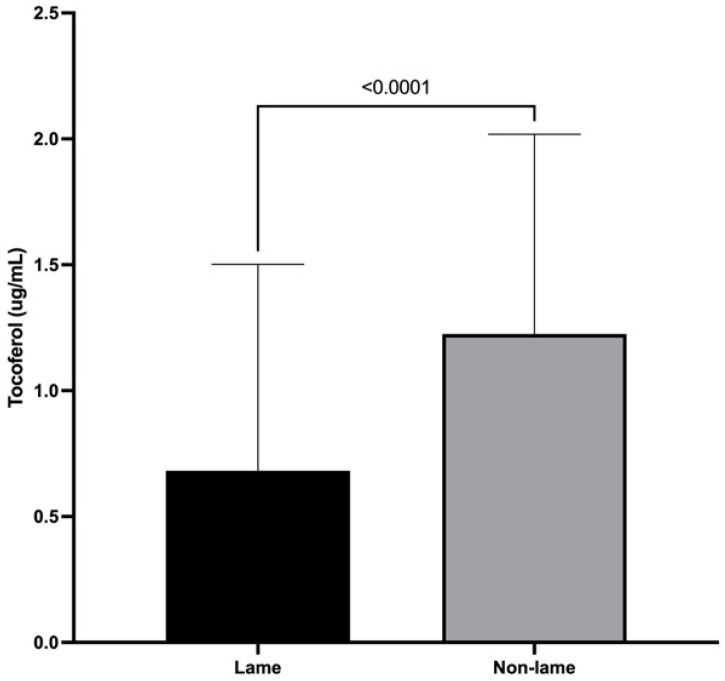
Mean + SD spinal cord concentrations of α-tocopherol of chronically lame and control dairy cows.

The mean spinal cord concentration of α-tocopherol was 0.68 ± 0.82 (μg/mL) for lame cows and 1.23 ± 0.79 for non-lame cows. The α-tocopherol spinal cord concentration was decreased in the lame group when compared with that of the non-lame group (*p* < 0.0001) (Figure 4).

## 4. Discussion

This study looks forward for new options for approaching the pathophysiology of lameness in cattle as well as new targets for the treatment of chronic pain using cattle as a non-induced translational model.

The results of this study showed an increased concentration of SP in the spinal cord of lame cows. The role of SP in chronic pain has been associated with its release from primary afferent fibers during inflammation, leading to upregulation of NK1 receptors in dorsal horn neurons [36]. Additionally, increases in SP/NK1 expression have been associated to microglial activation and NMDA receptor phosphorylation, both well-known events involved in central sensitization and chronic pain [26]. The activation of the NK1 receptor mediated by SP leads to increased glutamatergic transmission in the dorsal horn, spinal hyperexcitability, hyperalgesia, and allodynia [37]. Mechanical hyperalgesia and allodynia have been confirmed in lame dairy cows using mechanical algometry [4], and it is well known that higher expression of SP in the spinal cord induces nociceptive sensitization, hyperexcitability, and hyperalgesia, conditions that have been associated with the development of chronic pain [38]. Central sensitization has also been described in lame cows, observing a hyperalgesic response to measurements of the pain threshold [39,40] represented as a threshold reduction in nociceptive neuron activation [41].

Actual evidence supports the hypothesis that chronic pain is partially mediated by a dysregulation of endogenous endorphin signals [42]. Here, spinal BE showed increased concentrations in the lame group compared to those in the non-lame group, contrary to plasma BE concentrations reported in lame dairy cows [2]. Considering that chronic lameness represents a chronic pain state [4], the mechanism behind the impaired response of the endogenous opioid system could be due to sustained release of BE in earlier stages that leads to desensitization of the μ opioid receptor and the subsequently negative feedback of BE release [32], suggesting that BE release in lame cows is affected by the chronicity of the painful stimulus.

Furthermore, the continuous release of endorphins can induce long-lasting and latent pain hypersensitivity through a NMDA-dependent process, resulting in an exaggerated pain response to a further noxious stimulus [43]. Due to the aforementioned, we suggest that the spinal cord concentration of β-endorphin observed in this study could be a consequence of a defective response of the endogenous opioid pathway, which may represent a dysfunction of the descending modulatory pathway that produces reduced inhibition/enhanced facilitation, resulting in enhanced pain observed in many chronic pain conditions [38]. 

Decreased disulfide levels were observed in the spinal cord of chronically lame dairy cows compared with those of non-lame cows. Dynamic thiol–disulfide homeostasis represents an image of the oxidative stress condition and is a novel method for oxidative stress evaluation [14,15,16,17]. Our results indicate that thiol/disulfide homeostasis had shifted to the reduction phase as there was increased reduction of disulfides to thiols [44]. This could represent a solid response to the increased augmented ROS activity described recently [3] in dairy cows with chronic inflammatory lameness, confirming the potential effect of oxidative signaling over the regulation of several mechanisms involved in central sensitization [3]. The altered thiol/disulfide homeostasis observed in this study is also in agreement with previous reports that have associated abnormal thiol/disulfide homeostasis with the oxidative stress-related pathogenesis of various chronic conditions and diseases including diabetes [45], cancer [46], Parkinson’s disease [47], and fibromyalgia [44].

α-tocopherol is considered the major lipid-soluble antioxidant, preventing oxidative damage of membrane lipids by scavenging free radicals [20]; however, our results showed that α-tocopherol concentrations were also reduced in the spinal cord of lame dairy cows, which could represent an ongoing oxidative reaction [48]. This assumption agrees with the results presented recently [3] showing that cows with chronic lameness have increased levels of ROS and increased levels of lipid and protein oxidation. Moreover, it has been described that the treatment with α-tocopherol prevents changes in oxidative stress status in rats with neuropathic pain related to antioxidants effects [49]. Interestingly, this discrepancy could indicate a relation between the increased ROS levels described previously [3] and the decreased α-tocopherol concentrations in chronically lame cows suggesting an impaired antioxidant response.

Central sensitization is an inherent feature of chronic pain, and its development has been associated to increased ROS and RNS in experimental models of neuropathic and inflammatory pain [5,50]. The decreased concentrations of α-tocopherol and disulfide levels along with the increased concentrations of substance P and β-endorphin observed in our study suggest that chronic pain development was associated with increased ROS.

Some limitations in our study include a low number of cows, which can increase the chance for an associated beta error, decreasing the power of the statistical tests. Additionally, euthanasia has been indicated as a factor that could modify plasma biomarkers of pain. Nonetheless, no information regarding livestock could be found. Furthermore, the use of different methods of euthanasia between the groups could have caused unquantifiable spontaneous changes in the study variables. Despite several limitations, the results suggested that early increases of SP and BE could facilitate pain detection in lame cows and allow for newer pharmacological approaches to control lameness-associated pain in dairy cows.

## 5. Conclusions

In conclusion, the results presented for disulfide levels and α-tocopherol concentrations indicated a non-enzymatic antioxidant response to an oxidative stress state in cows with chronic lameness. Otherwise, the results of SP and BE concentrations indicated a chronic pain process as there was a direct relation of SP with microglial activation and pain maintenance, and an impaired endogenous analgesic response due to pain-induced long-lasting BE release.

## Data Availability

Data is available upon request at hbustamante@uach.cl.
